# Usage of extruded diamond multi-injectors for improvement of fuel mixing inside the supersonic combustion chamber

**DOI:** 10.1038/s41598-023-42487-2

**Published:** 2023-09-16

**Authors:** Hossein Seraj, Farhad Hosseinnejad, Yasser Rostamiyan, Keivan Fallah

**Affiliations:** Department of Mechanical Engineering, Sari Branch, Islamic Azad University, Sari, Iran

**Keywords:** Aerospace engineering, Mechanical engineering

## Abstract

The main attention of this work is to investigate the usage of diamond multi extruded injectors on the fuel distribution in combustor of scramjet. This study applied the computational technique to simulate the transverse fuel jets released from extruded nozzles. The main focus is to evaluate the role of induced shock waves on the penetration and distribution of fuel jets. The effects of jet space and usage of annular nozzle for the fuel injection system are revealed. Results of this work shows that the gap of jet would be more efficient for mixing when the inner air jet is also used. Also, injection of the air from the core of annular nozzle significantly increase the fuel mixing.

## Introduction

Fuel injection systems play a crucial role in achieving efficient fuel mixing in scramjets. Scramjets, or supersonic combustion ramjets, are air-breathing engines designed to operate at hypersonic speeds. These engines rely on the combustion of a fuel–air mixture to generate thrust. Efficient fuel mixing is essential to ensure proper combustion and maximize the engine's performance^1–3^.

Fuel injection systems for scramjets are responsible for delivering fuel into the combustion chamber in a controlled and efficient manner^4,5^. The primary objective is to achieve thorough and rapid mixing of the fuel with the incoming air, promoting stable combustion and optimal performance^6,7^. Various techniques and injector designs have been developed to address the unique challenges associated with scramjet fuel injection^8,9^.

The single-element injector is one of the simplest and most commonly used types of injectors in scramjet engines^10–13^. It consists of a single fuel injector element that injects fuel into the airflow. While simple in design, it may suffer from challenges such as uneven fuel distribution and limited control over the fuel–air mixing process^14–16^. However, it is a cost-effective solution that can be used in certain operating conditions^17^.

The multi-element injector incorporates multiple fuel injector elements arranged in a specific pattern^18–20^. Each element is responsible for injecting fuel at a particular location within the combustion chamber. This design allows for better control over the fuel distribution and mixing, resulting in improved combustion efficiency^21–24^. Multi-element injectors can be tailored to match specific flow conditions, making them suitable for a wide range of operating regimes^25,26^.

Impinging injectors utilize a series of fuel jets that impinge on each other or on an opposing surface, such as a fuel plate or a wall^27,28^. This design promotes intense fuel atomization and mixing due to the collision and breakup of the fuel streams. Impinging injectors offer enhanced fuel–air mixing and improved combustion stability^29–32^. However, they may be more complex to manufacture and require careful design considerations^33^.

The shear coaxial injector employs an inner fuel stream surrounded by an outer air stream. The fuel and air streams are forced to move in opposite directions, creating a shear layer between them^34,35^. This shear layer enhances fuel atomization and mixing, leading to improved combustion efficiency. Shear coaxial injectors are known for their ability to achieve high fuel–air mixing rates and can withstand a wide range of operating conditions^36,37^.

Air-blast injectors utilize compressed air to atomize and mix the fuel. The fuel is injected into a chamber where high-velocity air jets break it into small droplets, promoting rapid mixing with the incoming air. Air-blast injectors offer good control over fuel atomization and can provide efficient fuel–air mixing. However, they require a separate compressed air supply, which adds complexity to the overall system^38,39^.

Efficient fuel mixing is crucial for achieving optimal combustion and performance in scramjet engines. Various types of conventional injectors, including single-element injectors, multi-element injectors, impinging injectors, shear coaxial injectors, and air-blast injectors, have been developed to address the challenges associated with fuel injection in scramjets. Each injector type offers unique advantages and disadvantages, and the selection depends on factors such as operating conditions, performance requirements, and manufacturing considerations. Further research and development in fuel injection techniques continue to drive improvements in scramjet technology, aiming for enhanced fuel efficiency and propulsion performance.

**Figure 1:**
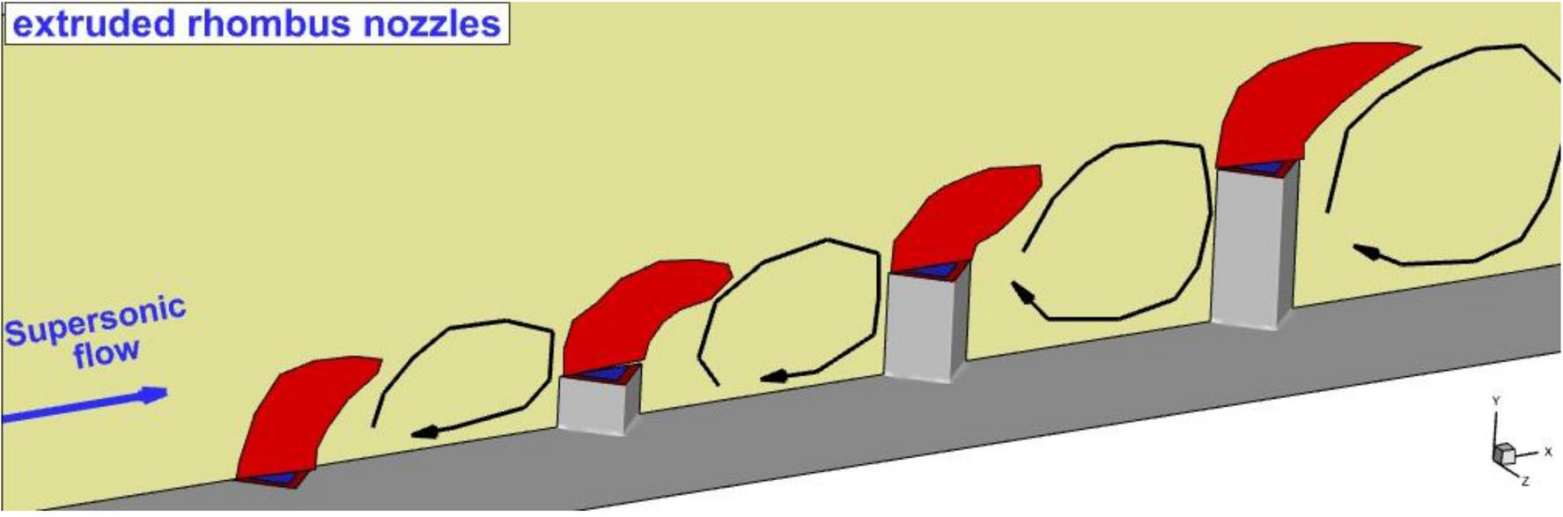
Proposed jet configuration.

This study has tried to present the mechanism of the fuel mixing of multi-diamond extruded nozzles inside the combustion chamber of the scramjet engine. The influence of jet configuration and the jet spaces are thoroughly analyzed in this article (Fig. 1). The CFD computational technique is used to model the supersonic compressible flow with transverse diamond extruded nozzles. The influence of coaxial air and fuel jets is also studied in this study.

## Governing equations and computational approaches

Computational fluid dynamic as a robust methodology is applied to model the mixing of the hydrogen multi jets in a combustion chamber. Since this study has tried to investigate the role of the fuel jet interaction on fuel mixing, RANS equations have been selected as the primary governing equations for the introduced model. For the simulations, energy equations were also solved instantly owing to the existence of the shock wave in our case. The SST turbulence model is also used owing to the high turbulence structure of the supersonic flow encounter jets and the extruded nozzle. The reactions are not considered in this model since their impacts on fuel diffusion are highly limited. Species mass transport equation is also considered for secondary gas which is hydrogen in the present study. Theoretical approach is widely used in mechanical engineering and science^40–46^.

**Figure 2:**
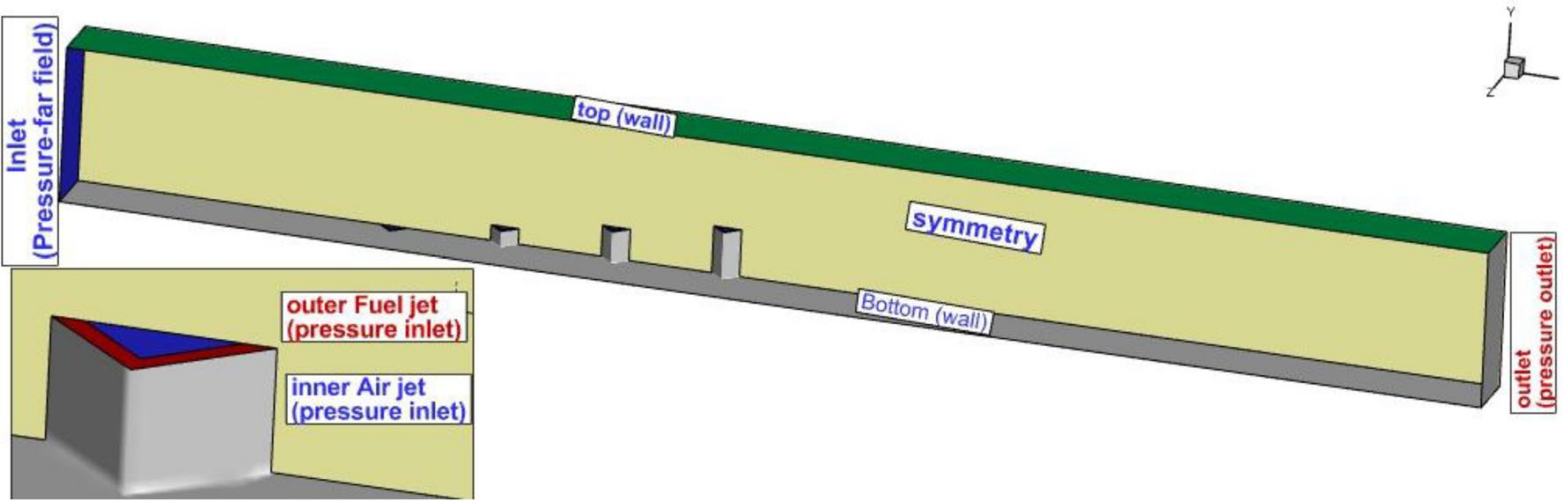
Applied boundary condition.

The selected geometry of the proposed multi-diamond extruded nozzles inside the combustion chamber of the scramjet engine is demonstrated in Fig. 2. The inlet flow with Mach = 4 and atmospheric pressure is applied on the inlet plane and the first extruded multi-diamond extruded nozzle is located 20 mm downstream of the inlet. The area of the fuel outer jet and inner air jet is equivalent to a circle with a diameter of Dj = 0.5 mm. The length of the domain is 100 mm and the depth is 1.5 mm. The height of the 1st, 2nd, 3rd, and 4th diamond extruded nozzles is 0, 0.5 mm, 1 mm and 1.5 mm. The fuel and air jet are released with a total pressure of 10% of free stream and sonic velocity. Two gap spaces (3 Dj and 7Dj) of the multi-diamond extruded nozzle's space are simulated in this research. More details on applied boundary condition is displayed in Fig. 2.

**Figure 3:**
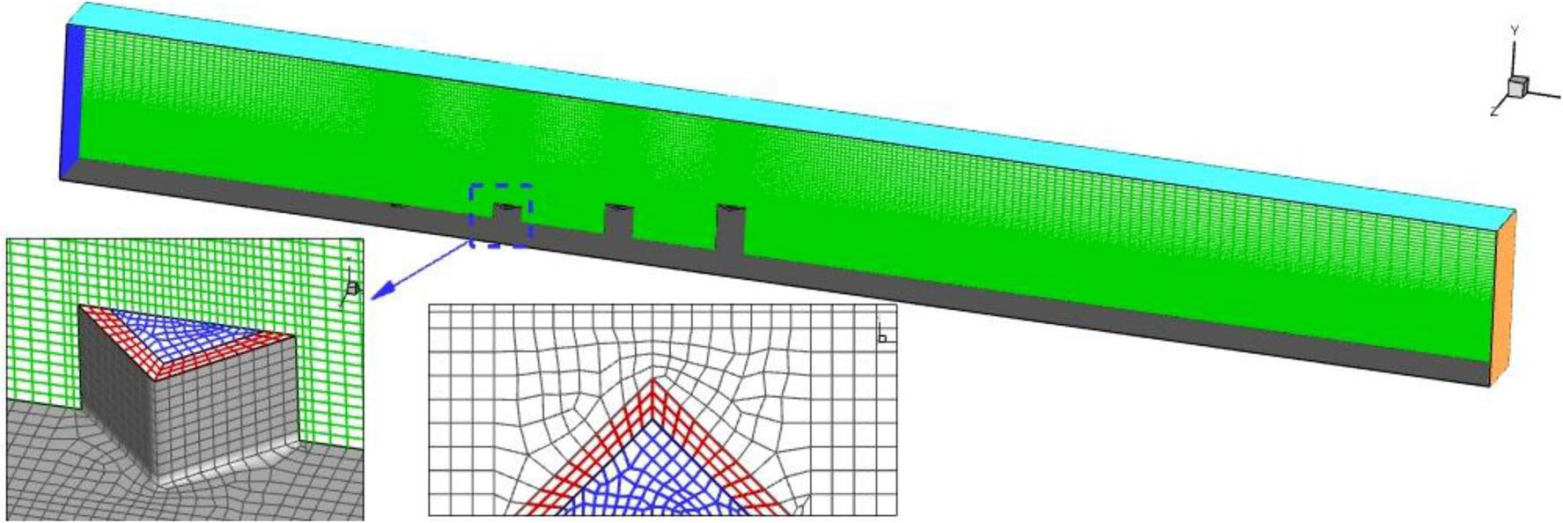
Grid production.

The generation of the grid is also done with specific characteristics related to the physics of the presented model. The structured grids are produced for the clarified model while its resolution is not uniform in the whole domain. As displayed in Fig. 3, the size of the grid near the extruded diamond injector is lower than other sections since the supersonic flow interactions with extruded nozzles and crossjets happen in this region^47–51^. Grid analysis is also performed to authenticate the results which should not be related to the size of the grids. In Table 1, average mass concentrations of produced grids are compared for the four generated grids. Thus, the fine grid is applied for future investigations.

**Table 1:** Grid study.

	Cells	Grid cells. along X, Y and Z direction	Hydrogen fraction (at 30 mm downstream)
Coarse	1,043,000	194 × 100 × 50	0.282
Medium	1,920,000	204 × 130 × 60	0.301
Fine	2,623,000	234 × 160 × 70	0.307
Very fine	4,200,000	251 × 200 × 80	0.308

## Results and discussion

The first step is to validate the results with other data to ensure the correctness of the simulations. In this work, the penetration value of the single jet is compared with experimental results in Table 2. The deviation of the computational results from experimental data is also defined in Table 2. The average deviation of the penetration height of a single circle nozzle from experimental data is less than 8% for the different locations downstream of the fuel nozzles.

**Table 2:** Validation (penetration height).

Distance from first jet (mm)	Pudsey et al.^39^ (mm)	Our data (mm)	Discrepancy (%)
5	6	6.5	8.3
10	7.1	7.5	5.6
20	7.6	7.9	3.9
30	8.1	8.4	4.1

**Figure 4:**
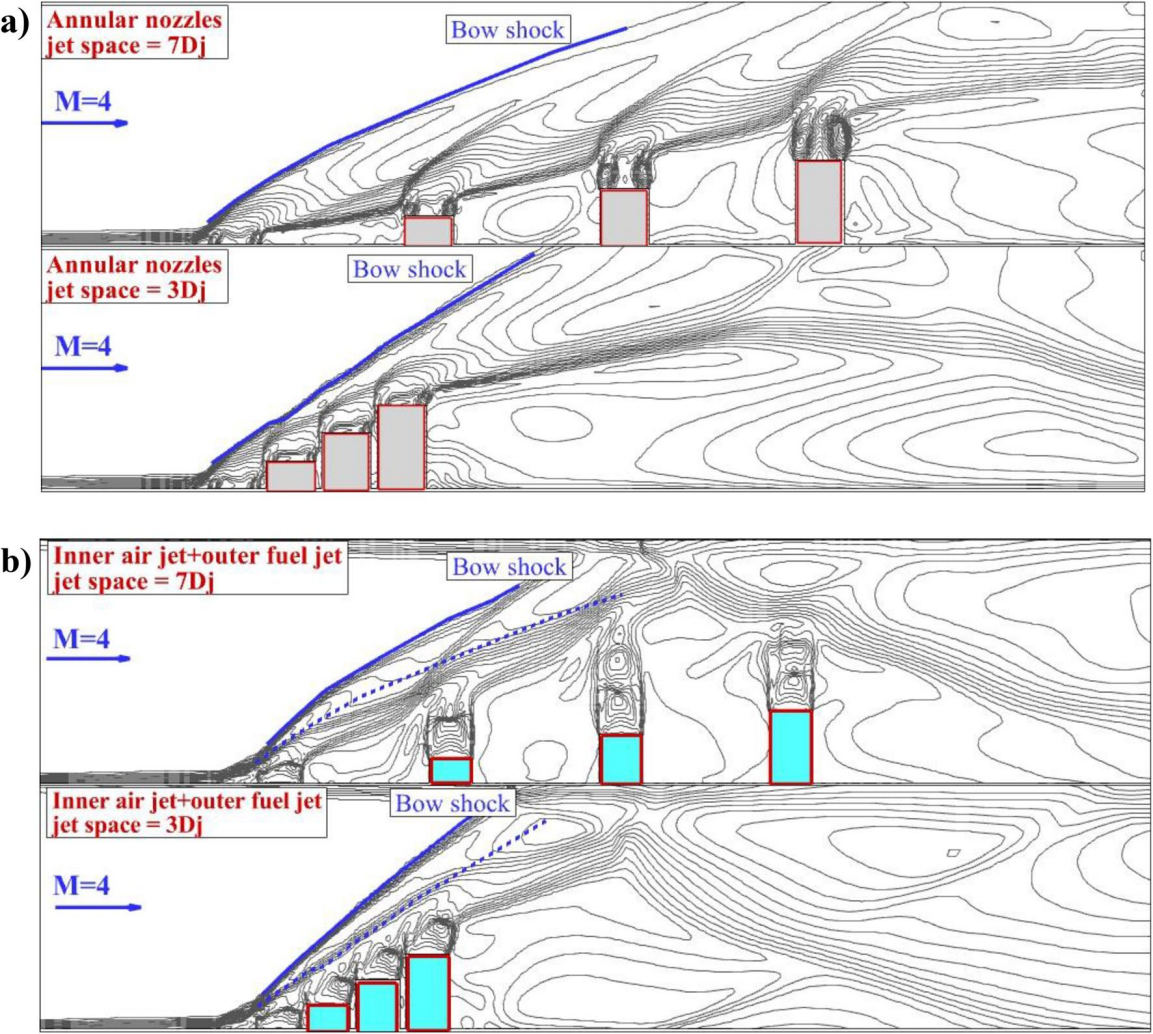
Mach contour on the symmetry plane (**a**) annular (**b**) coaxial jets.

Figure 4 illustrates the Mach contour on the symmetry plane of the annular nozzle for two jet spaces of 3Dj and 7Dj with/without inner air jets. In the annular injection model (Fig. 4a), the strong bow shock is noticed in front of the first jet and its angle is inherently related to the jet spaces. As noticed in the figure, tiny barrel shocks are generated near the injector and they are connected via a shear layer. The deflection of these barrel shocks near the nozzle is limited after 2nd extruded nozzle. As the jet spaces are reduced, the deflection of the produced barrel shock in the vicinity of the nozzles is more considerable. In Fig. 4b, the injection of the air jet from the inner core of the diamond nozzle increases the angle of the bow shock and this effect is visibly observed on the deflection of the barrel shocks produced by the annular fuel jets. Comparison of the bow shock for the annular jet (dashed blue line) with coaxial fuel and air jets (solid blue line) confirms the important effects of the inner air jets on the jet interactions.

**Figure 5:**
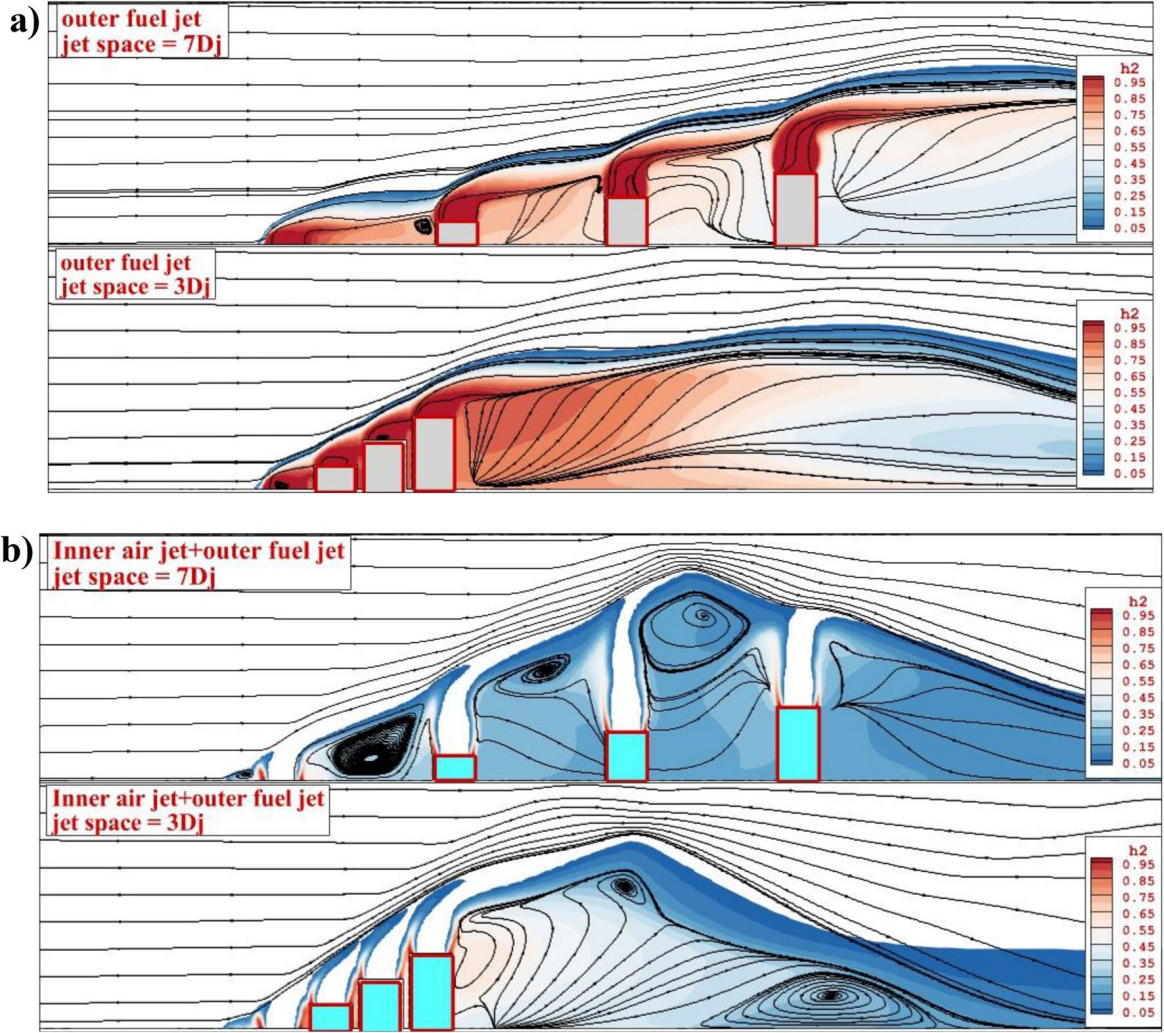
Comparison of mixing zone and streamline on jet plane (**a**) annular (**b**) coaxial jets.

Figure 5 demonstrates the influence of the jet space and fuel and air jet configurations on the fuel concentrations behind the jets. In Fig. 5a, the fuel mixing of annular extruded diamond nozzles for two jet spaces is illustrated. Since the bow angle of the case with lower jet spaces is higher than a model with jet spaces of 3Dj, the height of the fuel mixing zone is higher and consequently, the variation of the hydrogen concentrations decreases smoothly in low jet space. The flow circulation in the gap of the nozzle is also noticed in the model with a jet space of 7Dj. The addition of the inner air jets (Fig. 5b) totally changes the mass distribution in the combustion chamber and strengthens the role of the circulations. Since the injection of the inner air jet boosts the normal momentum of the fuel jet, the height of the penetration substantially increases and consequently, larger circulation is observed in the combustor. When the jet space is lower (3Dj), a single large circulation is noticed behind the last injector. However, there are several circulations in the jet gap when the jet space is increased.

**Figure 6:**
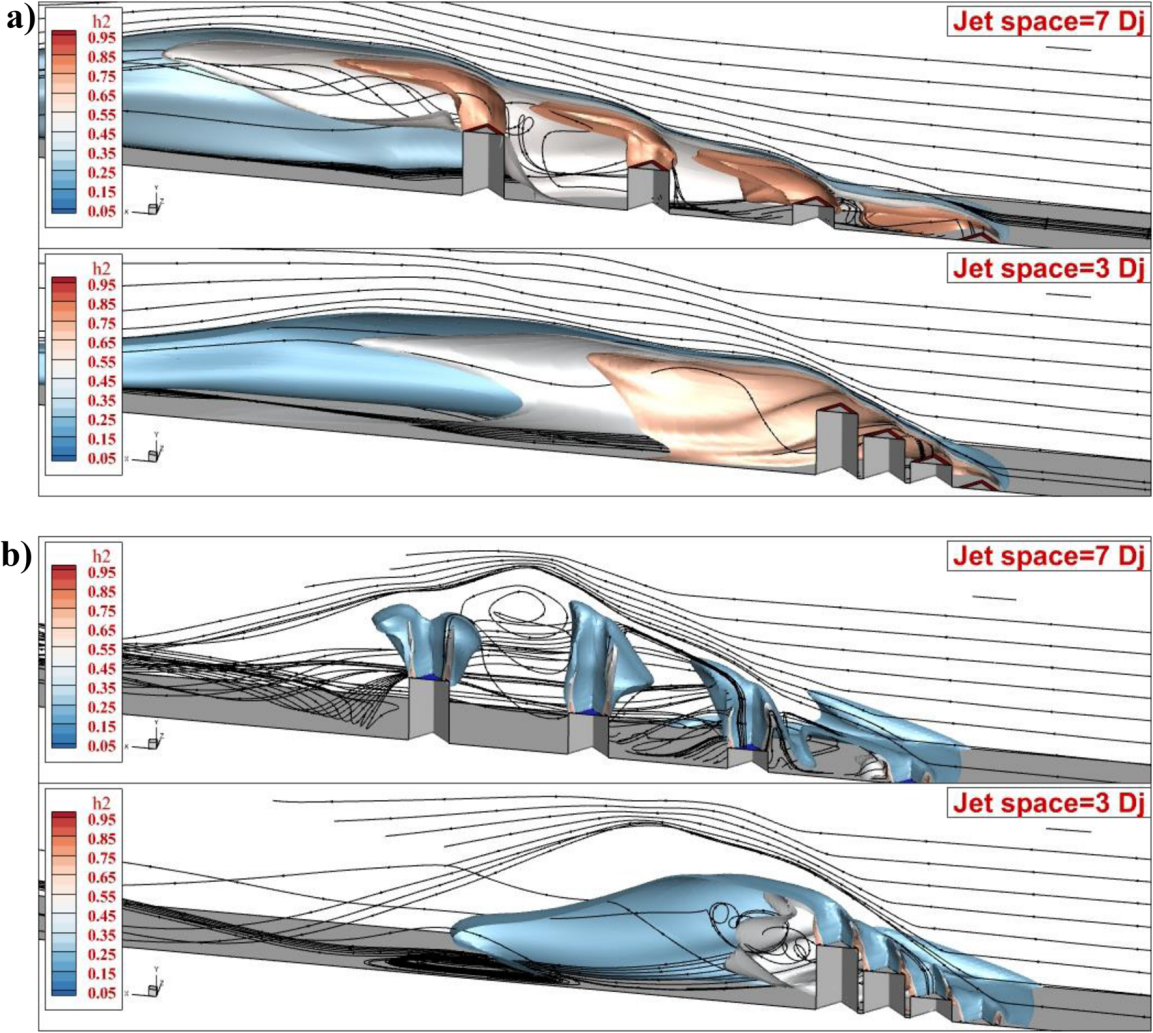
Comparison of 3-D feature of fuel jet (**a**) annular (**b**) coaxial jets.

The three-dimensional structure of the jet interactions is demonstrated in Fig. 6 for the proposed jet configurations. In the annular configurations, the produced vortex in the gap of the model with a higher jet pace (7Dj) confirms that the fuel jets diffuse more in the depth of the domain. The stream lie of the airflow has more interaction in this case. However, the main effective mechanism for the fuel mixing of the jet with a lower gap is the large circulation produced behind the last jet. The addition of the inner air jet entirely changes the core of the fuel jet in the model with a jet space of 7 Dj. The jet configuration in this model becomes independent and the airflow in the gap distributes fuel in the combustor. Now, it is valuable to display the mechanism of the circulation in these four configurations.

**Figure 7:**
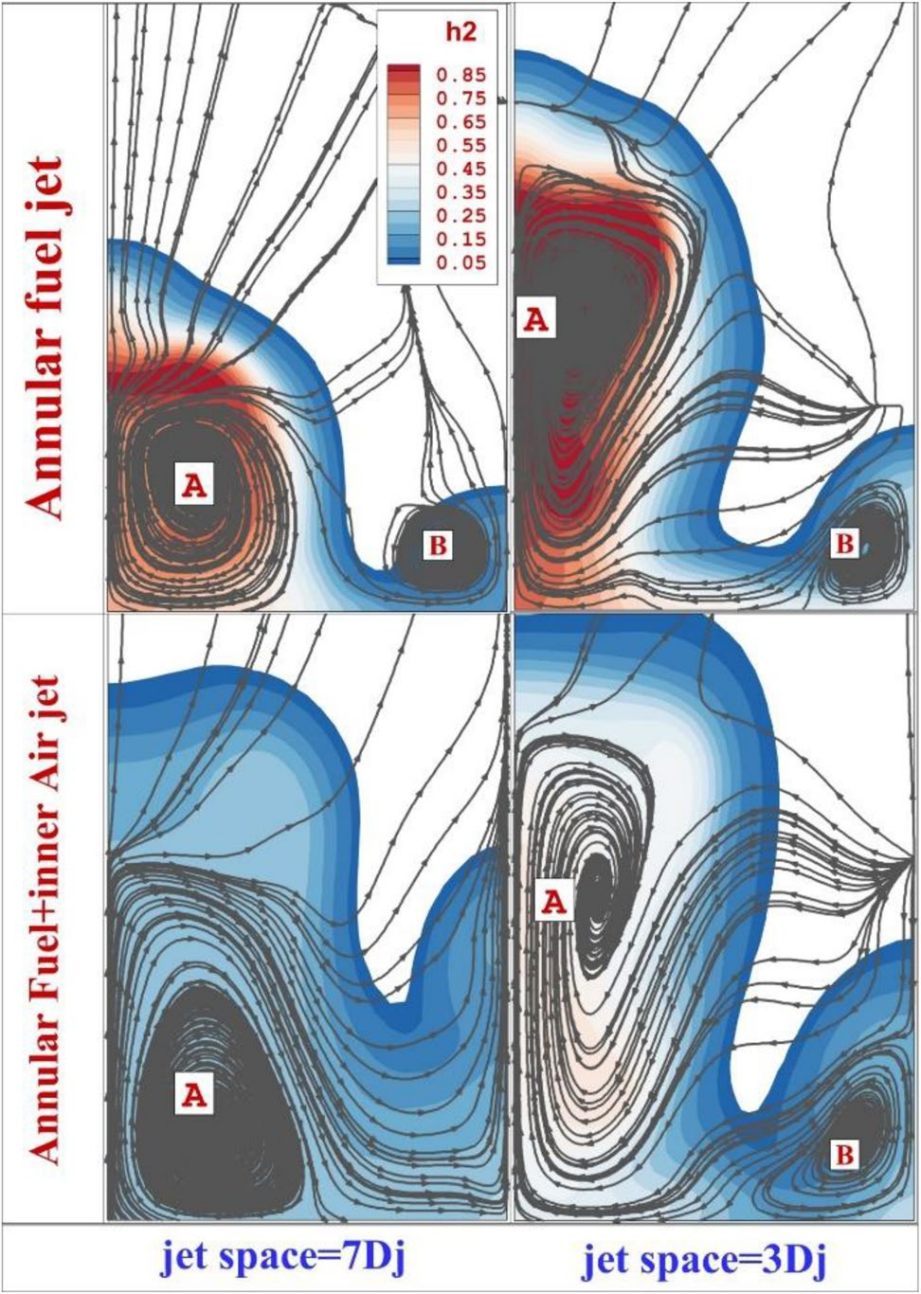
Comparison of mixing zone and streamline on plane 15 mm downstream of 1^st^ jet.

**Figure 8:**
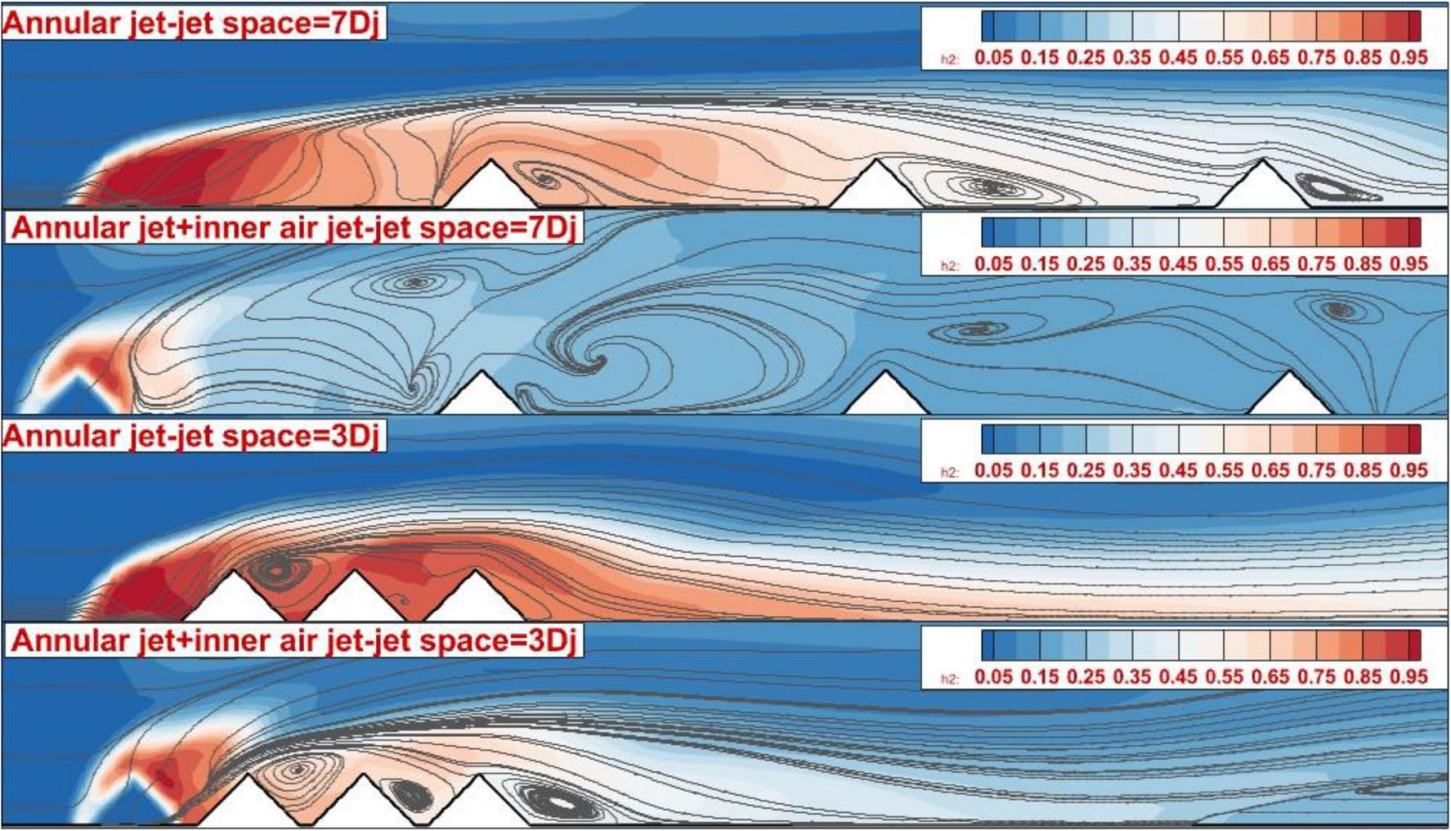
Comparison of mixing zone and streamline on plane 0.5 mm from bottom.

Figure 7 compares the mixing zone and flow stream of these jet configurations on fixed pale located 15 mm downstream of the first diamond jet. In the annular jet configurations, two main circulations are noticed. The first big one (A) is mainly due to the circulations while the second one is due to the horseshoe vortex occurring upstream of the first jet. As expressed before, the one with a lower jet space has higher fuel penetration and mixing zone near the injectors while the secondary vortex is the same for both jet spaces. When the inner jet is released from the inner nozzle, the hydrogen concentration drops behind the injectors while the mixing zone is expanded. In higher jet space the secondary vortex is disappeared while this is preserved when the jet space is 3Dj. Figure 8 illustrates the flow and fuel mass fraction on the plane located 0.3 mm from the bottom. The role of the air jet deflection induced by the extruded diamond nozzle on the hydrogen mass fraction and the flow stream is disclosed.

**Figure 9:**
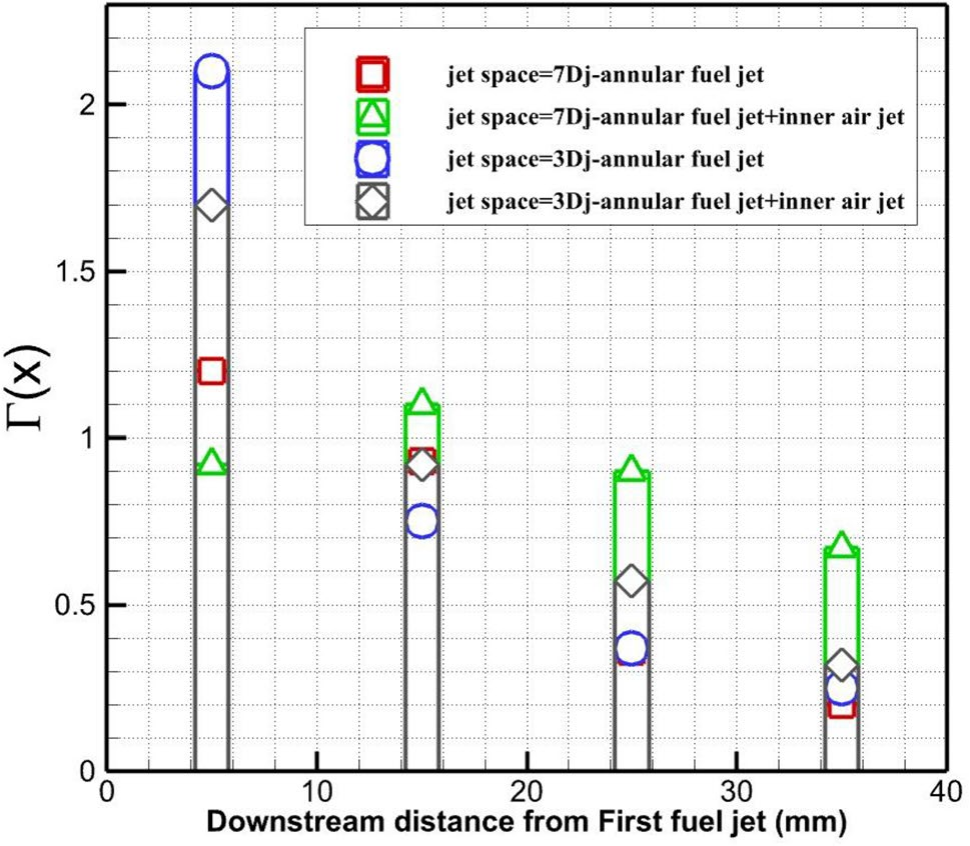
Variation of circulation behind the jets.

The distribution of the circulation strength behind the injectors is displayed in Fig. 9. Comparison of the annular injection system indicates that the lower jet space results in higher circulation strength near the nozzle while this strength power declines after 15 mm downstream. This pattern is preserved for coaxial air and fuel configurations. The strength of circulation is boosted by the usage of the inner air jet far downstream as demonstrated in Fig. 9. Besides, the achieved results show that the power of circulation decreased with a lower rate in the jet space 7Dj with coaxial configuration.

**Figure 10:**
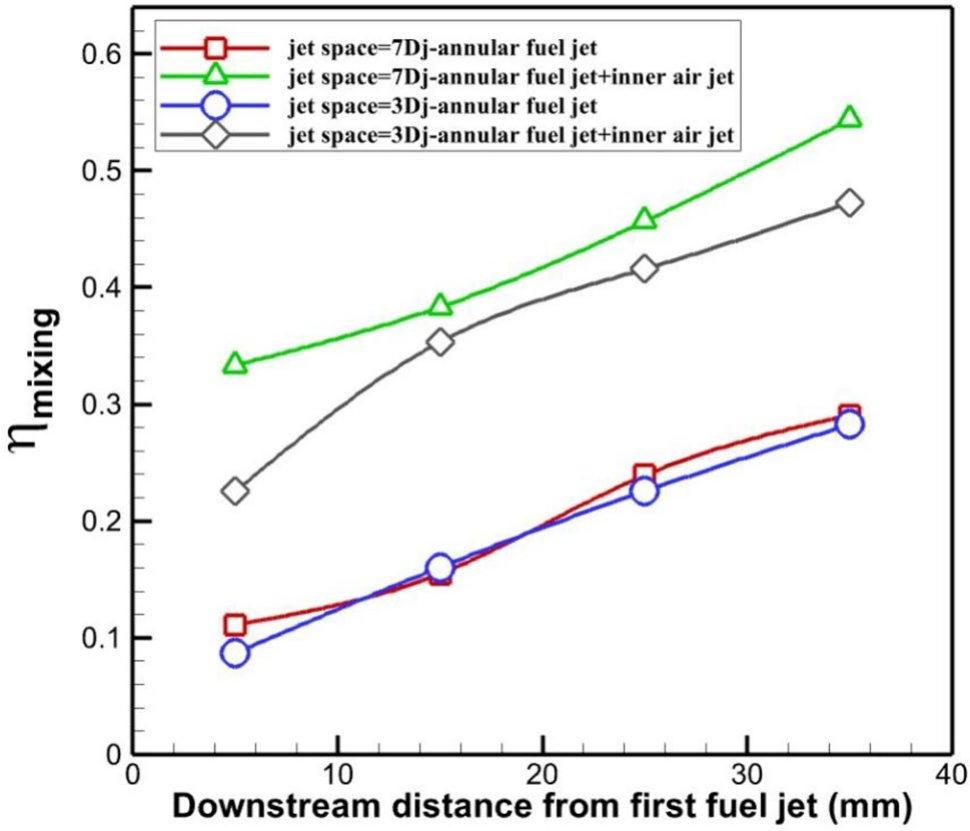
Variation of the fuel mixing behind the jets.

Figure 10 demonstrates the variation of the mixing efficiency for these four configurations. The plot shows that the mixing efficiency of the annular jet is almost identical while the use of inner air flow increases the fuel mixing up to 90% in jet space of 7Dj. However, the mixing performance boosts up to 75% in lower jet space. This confirms the role of vortex production by the usage of inner air flow.

## Conclusion

This study investigates the influence of the multi-diamond-shaped injectors on the mixing of hydrogen gas released inside the combustor. The usage of annular and coaxial jets is also studied in the present work. Computational fluid dynamics is applied for the modeling of the proposed injection system and discloses the fuel distribution mechanism in such complex flow physics. This work also studied the effects of fuel jet spaces on the vortex formation and consequently fuel mixing near the injectors. Mach contour analysis is also done to attain effective terms on the diffusion of the fuel in proposed injector configurations. The achieved outcome shows that the usage of the inner air jet improves the fuel mixing of the annular nozzle up to 90% downstream of injectors. In addition, higher jet space is more preferable since the vortex produced in the gap would preserve the fuel mixing efficiency downstream of the injector.

## Data Availability

All data generated or analysed during this study are included in this published article.
